# Atosiban versus placebo in the treatment of threatened preterm birth between 30 and 34 weeks gestation: study protocol of the 4-year APOSTEL 8 follow-up

**DOI:** 10.1136/bmjopen-2023-083600

**Published:** 2024-07-18

**Authors:** Larissa van der Windt, Job Klumper, Emilie V J van Limburg Stirum, Janneke van 't Hooft, Madelon van Wely, Aleid G van Wassenaer-Leemhuis, Eva Pajkrt, Martijn A Oudijk, APOSTEL 8 Study Group

**Affiliations:** 1 Obstetrics and Gynaecology, Amsterdam UMC Locatie AMC, Amsterdam, The Netherlands; 2 Amsterdam Reproduction and Development research institute, Amsterdam, Netherlands; 3 Netherlands Satellite of the Cochrane Gynaecology and Fertility Group, Amsterdam University Medical Centres, Duivendrecht, The Netherlands; 4 Department of Neonatology and Paediatrics, Emma Children's Hospital, Amsterdam UMC Location AMC, Amsterdam, The Netherlands; 5 Obstetrics, Amsterdam UMC Locatie De Boelelaan, Amsterdam, The Netherlands; 6 NVOG Consortium, Holland, The Netherlands

**Keywords:** Pregnant Women, Follow-Up Studies, OBSTETRICS

## Abstract

**Introduction:**

Currently, the majority of women worldwide with threatened preterm birth are treated with tocolytics. Although tocolytics can effectively delay birth for 48 hours, no tocolytic drug has convincingly been shown to improve neonatal outcomes and effects on long-term child development are unknown. The aim of this follow-up study of a placebo controlled randomised trial is to investigate the long-term effects of atosiban administration in case of threatened preterm birth on child’s neurodevelopment and behaviour development, overall health and mortality.

**Methods and analysis:**

This protocol concerns a follow-up study of the multicentre randomised double-blind placebo controlled APOSTEL 8 trial (NL61439.018.17, EudraCT-number 2017-001007-72). In this trial, women with threatened preterm birth (between 30 and 34 weeks of gestation) defined as uterine contractions with (1) a cervical length of <15 mm or (2) a cervical length of 15–30 mm and a positive fibronectin test or (3) in centres where cervical length measurement is not part of the local protocol: a positive fibronectin test or Actim-Partus test or (4) ruptured membranes, are randomised to atosiban or placebo for 48 hours. The primary outcome is a composite of perinatal mortality and severe neonatal morbidity. Children born to mothers who participated in the APOSTEL 8 study (n=760) will be eligible for follow-up at 4 years of corrected age and assessed using four parent-reported questionnaires. Primary outcomes are neurodevelopment and behaviour problems. Secondary outcomes are on child growth and general health. All outcomes will be compared between the atosiban and placebo group with OR and corresponding 95% CI. Analyses will be performed using the intention-to-treat approach.

**Ethics and dissemination:**

The Medical Research Ethics Committee from Amsterdam UMC confirmed that de Medical Research Involving Human Subjects Act (Dutch WMO-law) did not apply to our study (W21_386 # 21.431). Results will be published in a peer-reviewed journal and shared with stakeholders and participants. This protocol is published before analysis of the results.

**STRENGTHS AND LIMITATIONS OF THIS STUDY**

- This study will be a long-term follow-up study of the multicentre randomised double-blind placebo controlled APOSTEL 8 trial.
- This study will evaluate the effect of atosiban versus placebo in threatened preterm birth between 30 and 34 weeks of gestation on long-term child outcomes at 4 years of age concerning overall health, neurodevelopment and behaviour.
- For this follow-up study, three validated questionnaires will be used to evaluate child outcomes, including the Ages and Stages Questionnaire-3, Strengths and Difficulties Questionnaire and Vineland screener in combination with a questionnaire on overall health.
- Although less accurate than face-to-face evaluation, these parental questionnaires are useful as screening tools to detect child development and behaviour problems.

## Background

 In 2018, the WHO reported that preterm birth is the leading cause of death worldwide for children under the age of 5 years.^1^ Although various pharmacological agents and therapeutic interventions have been developed to reduce occurrence of preterm birth, preterm birth rates remain high.^2 3^ Apart from mortality, preterm birth can lead to both severe short-term and long-term neonatal morbidity, including neurodevelopmental disorders, respiratory problems and impaired ophthalmological functions.^4^,^7^

In case of threatened preterm birth, administration of tocolytics drugs is widely spread and has been part of standard care in many countries for decades. Treatment with tocolysis can delay imminent preterm birth to allow a complete course of antenatal corticosteroid administration hereby promoting fetal lung maturation and facilitating transfer to a hospital with neonatal intensive care unit (NICU) facilities.^8^,^10^ Various tocolytic drugs have shown to be effective in delaying delivery, including atosiban, however, no tocolytic drug has proven to be effective in reducing neonatal morbidity and mortality compared with a placebo.^11 12^ Therefore, the current use of tocolytic drugs in case of threatened preterm birth is insufficiently supported by available evidence. For this reason, our research group started the double-blinded randomised controlled Assessment of Perinatal Outcome after Specific Tocolysis in Early Labour (APOSTEL) 8 trial in 2017. This trial evaluates the effectiveness of atosiban versus placebo administered for 48 hours in women with a diagnosis of threatened preterm birth between 30 and 34 weeks of gestation on neonatal morbidity and perinatal mortality.^13^

Besides the effects on the immediate neonatal period before hospital discharge, it is essential to extend our scope on long-term child’s health and developmental outcomes since pregnancy interventions can have unexpected harmful long-term effects.^14^,^18^ However, thus far only a few randomised trials concerning tocolytic drug administration during pregnancy have performed long-term follow-up on child development. Our research group conducted the APOSTEL II trial, comparing nifedipine maintenance therapy to placebo in case of threatened preterm birth between 26 and 32 weeks of gestation.^19^ At 2 years of age, we found a higher rate of fine motor problems in the nifedipine group (n=78) (22.2% vs 7.6%), but lower rate of poor problem-solving (21.2% vs 29.1%) compared with the placebo group (n=66).^20^ We concluded that there was insufficient evidence to support use of nifedipine for maintenance tocolysis. Second, the APOSTEL III trial compared tocolysis with nifedipine versus atosiban in threatened preterm birth between 25 and 34 weeks of gestation.^21^ Follow-up at 2.5–5.5 years showed composite abnormal development in 30% of children in the nifedipine group (n=115) and 38% in the atosiban group (n=110).^22^ Neither nifedipine nor atosiban was considered as preferred treatment in women with threatened preterm birth on both short-term and long-term outcome. A recent population-based cohort study including 600 children reported no difference in neurodevelopmental outcomes at 5.5 years among children with and without antenatal exposure to tocolysis by atosiban or nifedipine. However, follow-up outcomes were retrospectively obtained and the population solely concerned pregnancies complicated by preterm prelabor rupture of membranes between 24 and 32 weeks gestation.^23^

In conclusion, long-term effects of tocolytic drug administration during pregnancy on child outcomes are still largely unknown. We will, therefore, perform a follow-up study of the randomised placebo-controlled APOSTEL 8 trial. This follow-up study aims to determine the long-term effects of atosiban versus placebo in threatened preterm birth on neurodevelopment, behaviour problems and general health of children around 4 years of corrected age. The APOSTEL 8 follow-up will help to identify potential beneficial or harmful effects of atosiban administration during pregnancy and will contribute to further improving threatened preterm birth healthcare.

## Methods and analysis

### Study setting

This study will be a follow-up study of the APOSTEL 8 study, an international, multicentre, randomised, double-blinded, placebo-controlled trial performed across 30 hospitals situated in the Netherlands, UK and Ireland (NL61439.018.17, EudraCT-number 2017-001007-72). The APOSTEL 8 trial includes women with threatened preterm birth between 30 and 34 weeks of gestation. Threatened preterm birth is defined as uterine contractions with (1) a cervical length <15 mm; (2) a cervical length of 15–30 mm with a positive fetal fibronectin test; or (3) a positive fibronectin test or insulin-like growth factor binding protein-1 (Actim-Partus test) in centres where cervical length measurement is not part of local protocol or (4) ruptured membranes. Eligible women are randomised to atosiban or placebo (0.9% saline) for 48 hours. The primary outcome is a composite of adverse perinatal outcomes including perinatal mortality and six severe perinatal morbidities: bronchopulmonary, periventricular leukomalacia >grade 1, intraventricular haemorrhage >grade 2, necrotising enterocolitis ≥stage 2, retinopathy of prematurity >grade 2 or needing laser therapy, andculture-proven sepsis. Secondary outcomes include various infant and maternal outcomes which can be found in the published protocol. The APOSTEL 8 started in 2017 and complete recruitment in 2023. Long-term follow-up of the APOSTEL 8 study was announced in the original trial protocol.^13^ This long-term follow-up study is directed at the children born to mothers who participated in the APOSTEL 8 trial, therefore, either exposed to atosiban or placebo during pregnancy. We will use four parent-reported questionnaires to assess neurodevelopment, behaviour problems and general health. Data collected in the original trial will be linked to the data obtained in this follow-up study. The study protocol has been developed according to the Standard Protocol Items: Recommendations for Interventional Trials criteria.

### Participants/eligibility criteria

Children of both singleton and multiple pregnancies, born to mothers who participated in the APOSTEL 8 trial, will be eligible for inclusion. Assessment will be performed when children are around 4 years of corrected age (ideally between 3 years and 9 months (3;9) to 4 years and 3 months (4;3) years of corrected age). Participants who did not give consent to be approached for follow-up research in the original APOSTEL 8 trial, will not be approached in this follow-up study. Also, participants included in the UK or Ireland will not be eligible for inclusion.

### Study design

The APOSTEL 8 follow-up study will be performed within the Dutch consortium for Healthcare Evaluation and Research in Obstetrics and Gynaecology (NVOG Consortium; https://zorgevaluatienederland.nl/NVOG). Good clinical practice trained researchers from the Amsterdam University Medical Center will cross-check medical records and Dutch Personal Record database of both mother and child(ren) for the possible occurrence of death. Thereafter, information letters and informed consent forms of this follow-up study will be sent by email to the APOSTEL 8 participants. Contact details of the research team are shared in the information letter to facilitate the opportunity to ask potential questions or discuss concerns. If the email address is unknown, participants will be contacted by phone and informed consent forms will be sent by mail. After giving informed consent, participants are redirected to the online questionnaire via a web link. Participants can withdraw participation at any time.

#### Blinding

The APOSTEL 8 trial is a double-blind trial. Blinding will be retained in this follow-up study, both for patients and investigators. After completion of participation in this follow-up trial, participants will be offered information on their allocation.

### Outcomes

The primary outcomes of this follow-up study include a composite of children’s (mildly) abnormal neurodevelopment and/or behaviour problems. Secondary outcomes include child’s growth, weight and general health. We will report the outcomes as a separate outcome, as well as a composite outcome as described below. We will present data as continuous scores (with mean and SD or median with IQR) and/or as dichotomised scores (based on the predefined cut-off scores). See table 1 for an overview of the questionnaires and outcomes.

**Table 1:** Overview of outcomes

Outcome	Method of measurement	Definition	Outcome measurement
Neurodevelopment	Ages and Stages Questionnaire third edition (ASQ-3)	Scores on five domains: Communication Gross motor skills Fine motor skills Problem-solving skills Personal-social skills	Mean (SD)Abnormal:≥2 SD below mean in any domain≥1 SD below mean in multiple domains*Mildly abnormal:≥1 and <2 SD below mean in one domain*
	Vineland screener	Total adaptive functioning score based on four domains: Communication Social skills Daily living skills Motor skills	Mean (SD)Abnormal:≤10th percentile of the population*Mildly abnormal:11th–25th percentile of the population*
Behaviour	Strength and Difficulties Questionnaire (SDQ)	Total difficulties score including four subscales: Conduct problems Emotional symptoms Hyperactivity Peer relationships	Mean (SD)Abnormal:>90th percentile of the population*Mildly abnormal:80th–90th percentile of the population*
Mortality	Cross-check medical files and Dutch Personal Record database	Perinatal mortality and death up to around 4 years of corrected age	Number (%)†
General health	General Health Questionnaire‡	Height	Mean (SD)Abnormal: 1.6 SD above or below target height range*
		BMI	Mean (SD)Abnormal^34 35^: underweight* overweight* obesity*
		Morbidity, medication use, hospital admissions, surgeries	Number (%)
Primary outcome	ASQ-3, Vineland screener, SDQ and mortality	Composite outcome is divided into abnormal and mildly abnormal	Abnormal:Score in ASQ-3, vineland screener or SDQ questionnaire is abnormal as defined aboveMildly abnormal:Score in ASQ-3, vineland screener or SDQ questionnaire is mildly abnormal as defined above

* Cut-off according to age and gender.The denominator changes into all children born to participants of the original APOSTEL 8 trial.

† This questionnaire was developed by our research team that is specialised in follow-up research of obstetric intervention studies. The questionnaire has been used in multiple follow-up studies.The denominator changes into all children born to participants of the original APOSTEL 8 trial.

‡ This questionnaire was developed by our research team that is specialised in follow-up research of obstetric intervention studies. The questionnaire has been used in multiple follow-up studies.^22 30 38^

APOSTEL 8Assessment of Perinatal Outcome after Specific Tocolysis in Early Labour 8BMI, body mass indexSDS, SD Score

#### (Neuro)development

The third version of the Ages and Stages Questionnaire (ASQ-3) will be used to assess neurodevelopment. The ASQ-3 is a screening tool to monitor child development by measuring five domains: communication, gross and fine motor skills, problem-solving skills and personal-social skills and can be used till 6 years of age. The ASQ-3 is the most commonly used parent-reported screening tool on development worldwide and is validated in a Dutch reference population.^24^

Interpretation: scores of ≥1 SD below the mean of the ASQ normative data in two or more domains or ≥2 SD below the normative mean in at least one domain will be considered abnormal. A score of ≥1 and <2 SD below mean in one domain will be considered as mildly abnormal.

The Vineland screener is a tool developed in the Netherlands to assess adaptive functioning in children up to 6 years of age. Adaptive functions refers to how well a person copes with common demands in life. The tool consists of 72 questions concerning everyday behaviour and covers four domains: communication, social, motor and daily living skills. The total adaptive functioning score is the sum of these four domains.^25 26^

Interpretation: A score between the 11th and 25th percentile of the population is considered mildly abnormal (77–84 for children of 3–4 years of age and 100–107 for children 4–5 years of age). A score ≤10th percentile of the population is considered as abnormal (≤76 for children of 3–4 years of age, ≤99 for children of 4–5 years of age).

#### Behaviour problems

The Strengths and Difficulties Questionnaire (SDQ) will be used to assess behaviour disabilities. The SDQ is a screening tool to identify behavioural problems in children from 3 to 17 years of age and covers five domains: emotional symptoms, conduct problems, hyperactivity/inactivity, peer problems and prosocial behaviour. Apart from domain scores, a total difficulties score can be calculated including the first four domains described previously.^27 28^ The SDQ has been validated in a Dutch reference population.^29^

Interpretation: for children below 4 years of age, a Total Difficulty Score of ≥16 points is considered abnormal (>90th percentile) and a score of 13–15 points is considered mildly abnormal (80th–90th percentile). For children older than 4 years of age, a Total Difficulty Score of ≥17 is considered abnormal (>90th percentile) and a score of 14–16 points is considered mildly abnormal (80th–90th percentile).

#### Mortality

Child death (defined as perinatal mortality and death up to 4 years of corrected age). Medical records and the Dutch Personal Records Database will be used to verify the number of deceased children.

#### General health

We used a general health questionnaire to assess general health of children. This questionnaire was developed by our research team and has been used in several previous obstetric follow-up studies performed by the nationwide obstetric consortium.^30^,^32^ Questions concern child growth and health related problems (ie, need for surgery, hospital admissions, medication use and reported medical conditions).

#### Interpretation

- Growth: we will present height as a continuous and dichotomous outcome (normal/abnormal score). An abnormal score is defined as 1.6 SDS above or below target height range based on parental height Dutch reference values.^33^ We will calculate the body mass index (BMI) and will report BMI as a continuous outcome and as a proportion of children who are underweight, overweight or obese based on Dutch reference data.^34 35^
- Health-related problems: we will report on medical conditions, hospital admissions, medication (used) and history of surgery.

#### Composite outcomes

Since both behaviour and neurodevelopment are equally important, we report on a composite outcome. Composite adverse child outcome is defined as:

- Abnormal adverse child outcome: if the score in ASQ-3, Vineland screener or SDQ questionnaire is abnormal as defined before.
- Mildly abnormal adverse child outcome: if the score in ASQ-3, Vineland screener or SDQ questionnaire is mildly abnormal as defined before.
- Death or survival with abnormal cognitive and/or behaviour development: abnormal adverse child outcome as defined previously or death up to 4 years after randomisation.

### Sample size

The original APOSTEL 8 superiority trial with an aimed sample size of 760 pregnancies (of which approximately 635 singletons and 125 multiples). Since only participants of the original APOSTEL 8 trial are eligible for inclusion in this follow-up study, the maximum number of possible participants is fixed. Based on our previous follow-up studies using questionnaires, we expect to realise a follow-up rate of 50%,^20 22 36^ resulting in approximately 400 children to be included. Group sample sizes of 200 children in the atosiban group and 200 children in the placebo group achieve 80% power to detect a difference of 12% between group proportions in our composite main outcome. The test statistic used is the two-sided Z-Test with pooled variance with a significance level of the test is 0.05.

#### Statistical analysis

Differences in baseline characteristics of APOSTEL 8 follow-up participants will be compared between atosiban and placebo group using unpaired t-test, Mann-Whitney U test, χ^2^ test or Fisher’s exact test when appropriate. A two‐sided p<0.05 will be considered statistically significant. To detect potential attrition bias, baseline characteristics of follow-up participants will be compared with those lost to follow-up.

For the main outcomes, neurodevelopment and behaviour, we will report mean scores with SD and dichotomous outcomes (abnormal/mildly abnormal) of the domains and total score of the ASQ-3, Vineland screener and SDQ. For general health-related outcomes, we will report on the outcomes as previously described. For the outcome mortality, the denominator needs to be changed into all children born to participants of the original APOSTEL 8 trial.

For the main analysis, all outcomes will be analysed using generalised linear mixed effects model to enable accounting for the dependence of multiple pregnancies. For continuous outcomes, this will provide mean difference with 95% CI and for dichotomous outcomes OR with 95% CI. All analyses will be performed according to the intention-to-treat principle and complete case analysis using SPSS or R (latest version).

#### Additional sensitivity analyses

In accordance with the APOSTEL 8 trial, the following subgroup analyses are planned:

- Singleton and multiple pregnancy separately.
- Cervical length <15 mm vs cervical length 15–30 mm and a positive fibronectin test (or no

cervical length measurement and a positive fibronectin test or Partus test).

- Ruptured vs intact membranes at entry.
- Previous preterm birth.
- Excluding children that exceed the optimal window of 3;9 years–4;3 years of corrected age. In sensitivity analyses, we will use imputation techniques to impute missing outcome data for children lost to follow-up. Multiple imputation techniques will only be applied when it can be assumed that data are missing at random and the follow rate is >70%. the follow-up rate is lower than 70, a best-case and worst-case scenario analyses will be performed.
- Composite outcome of deceased children or survived children with abnormal cognitive and/or behaviour development. Combining mortality data with survival of children with a severe developmental disability will help in providing the full scope of relevant outcomes from the start of randomisation until up to 4 years of age. The denominator in this analyses will be all children born in the APOSTEL 8 trial, instead of the number of children included in follow-up. However, this outcome has some challenges due to the high probability of loss to follow-up (as is the case in many obstetrical follow-up studies after several years), and therefore, the inevitable need to deal with missing data. This will be done by either applying multiple imputation techniques or, in case of a high loss to follow-up, a worst-case and best-case scenario analyses.

#### Data management

To ensure confidentiality, participants of the original APOSTEL 8 trial will be registered pseudonymised using a six-digit subject identification code. Only if necessary, researchers are able to identify subjects by a code. Procedures of this follow-up study will all be in accordance with the Dutch Personal Data Protection Act. Questionnaire will be collected using the data management system Castor Electronic Data Capture. For this data collection, the subject identification code will be used.

#### Patient and public involvement

Late neurodevelopmental morbidity is one of the core outcomes identified in the core outcome set for studies evaluating interventions to prevent preterm birth.^37^ During the development of this core outcome set, patient representatives, members of patient organisations (eg, the European Foundation for the Care of Newborn Infants) and parents were actively involved. Furthermore, members of the Parents of Preterm Children Association (care4neo.nl) have stressed the importance of follow-up research. In 2017, 75 of their members filled in an online survey wherein 85% of the parents expressed to have concerns about their child’s long-term development. In their opinion, crucial outcomes to assess in long-term follow-up research are; child’s school attainment, cognitive development, behaviour and psychological problems, motor skills, respiratory problems, general health, growth and medication use. In 2019, our research team organised a focus group for women who delivered preterm, showing comparable results. Both the online survey and focus group have primarily determined our choice of outcomes in this follow-up study.

Two Dutch patient associations, the Vereniging van Ouders van Couveusekinderen (freely translated to Society of parents of children admitted to NICU) and the Nederlandse Vereniging van Ouders van Meerlingen (freely translated to Dutch society of parents of multiples) were involved in the design of the original APOSTEL 8 trial and will be updated on progress of the study and informed of study results.
