# Supplementary material for: Atosiban versus placebo in the treatment of threatened preterm birth between 30 and 34 weeks gestation: study protocol of the 4-year APOSTEL 8 follow-up
Source: BMJ Open. 2024 Jul 18;14(7):e083600. doi: 10.1136/bmjopen-2023-083600 (PMC11261668; doi:10.1136/bmjopen-2023-083600)
Supplement: Supplementary file 1 [file bmjopen-2023-083600.reviewer_comments.pdf]

## PEER REVIEW HISTORY

BMJ Open publishes all reviews undertaken for accepted manuscripts. Reviewers are asked to complete a checklist review form (**http://bmjopen.bmj.com/site/about/resources/checklist.pdf**) and are provided with free text boxes to elaborate on their assessment. These free text comments are reproduced below.

## ARTICLE DETAILS

| | |
|---|---|
| **TITLE (PROVISIONAL)** | Atosiban versus placebo in the treatment of threatened preterm birth between 30 and 34 weeks gestation – study protocol of the 4 year APOSTEL 8 follow-up |
| **AUTHORS** | van der Windt, Larissa; Klumper, Job; van Limburg Stirum, Emilie; van 't Hooft, Janneke; van Wely, MadelonEditorial Board Member; van Wassenaer-Leemhuis, Aleid; Pajkrt, Eva; Oudijk, Martijn; Study Group, APOSTEL 8 |

## VERSION 1 – REVIEW

| | |
|---|---|
| **REVIEWER** | Simon, Esfahan<br>Arba Minch University, Midwifery |
| **REVIEW RETURNED** | 19-Jan-2024 |

| | |
|---|---|
| **GENERAL COMMENTS** | #Your title should be SMART<br># make clear the abstract of the protocol.<br># How do you think about the feasibility of your study? |

| | |
|---|---|
| **REVIEWER** | Songthamwat, Metha<br>Department of Obstetrics and Gynecology, Udonthani Hospital |
| **REVIEW RETURNED** | 22-Jan-2024 |

| | |
|---|---|
| **GENERAL COMMENTS** | Background: no reference 1<br>Reference: reference 2 is not correct. |

| | |
|---|---|
| **REVIEWER** | Hu, Ying<br>Zhejiang University School of Medicine Women's Hospital |
| **REVIEW RETURNED** | 25-Jan-2024 |

| | |
|---|---|
| **GENERAL COMMENTS** | Thanks to the editor for the invitation. This is a study protocol evaluating the effect of atosiban versus placebo in threatened preterm birth between 30-34 weeks of gestation on long-term child outcome. Please see my comments below.<br><br>1.It's better to provide more details of threatended preterm birth, such as the definition of uterine contractions, and the exclusion of preterm birth.<br><br>2.Page 5 line 44 "However, thus far only few randomised trials concerning tocolytic drug administration during pregnancy have performed longterm follow-up on child development." The author then listed two trials of your own research group, APOSTEL II trial and APOSTEL III trial. Are there no other studies on atosiban?<br><br>3.How to consider covariates in statstical analysis, such as maternal age, educational level, etc. |

| REVIEWER | Liang, Zhaoxia<br>Zhejiang University, Obstetrical Department |
|---|---|
| REVIEW RETURNED | 25-Jan-2024 |

| GENERAL COMMENTS | Thanks for the invitation to review. This is a study protocol evaluating the effect of atosiban versus placebo in threatened preterm birth between 30-34 weeks of gestation on long-term child outcome. Please see my comments below.<br><br>1.Please clearly clarificate the reason for chosing threatened preterm birth between 30-34 weeks.<br>2.Page 5 line 27 “no tocolytic drug has proven to be effective in reducing neonatal morbidity and mortality compared to a placebo.” Dose this mean that these studies included atosiban, or that there are currently no studies of atosiban research?<br>3.Page 7 line 21 “The primary outcome is a composite of adverse perinatal outcomes including perinatal mortality before discharge from hospital. Secondary outcomes include various infant and maternal outcomes. ” It’s better to provide more details of these outcomes. |
|---|---|

**VERSION 1 – AUTHOR RESPONSE**

| Reviewer 1 | Mr. Tesfahun Simon, Arba Minch University |
|---|---|
| A. Comment 1 | Your title should be SMART |
| B. Response | Due to the nature of the study, we are bound to the title of the original APOSTEL 8 trial. |
| C. Changes made | To better meet the SMART objectives, we have specified at what gestational age women were included in the title. |
| D. Location | Page 1 |
| A. Comment 2 | Make clear the abstract of the protocol. |
| B. Response | The current abstract is reported according to the BMJ Open guidelines for a study protocol. We are unsure how to further clarify the abstract of our protocol. However, we did clarify our definition for threatened preterm birth in the abstract. |
| C. Changes made | We added our definition of threatened preterm birth. |
| D. Location | Page 2 |
| A. Comment 3 | How do you think about the feasibility |
| B. Response | Optimizing feasibility of long-term follow-up studies is a common challenge. To optimize the feasibility of our long-term follow-up study, we use digital parental questionnaires. In this way, we expect to contact a great majority of parents. Furthermore, through various patient organizations we are aware that parents find it important that long-term outcomes are assessed. We therefore expect most parents to be willing to fill out the questionnaires. We expect a follow-up rate of 50% based on previous follow-up studies |

| | |
|---|---|
| | (see methods section sample size page 11). |
| C. Changes made | None. |
| D. Location | - |

| | |
|---|---|
| Reviewer 2 | Dr. Metha Songthamwat, Department of Obstetrics and Gynecology, Udonthani Hospital |
| A. Comment | Background: no reference 1<br>Reference: reference 2 is not correct. |
| B. Response | Thank you for your precision, you are correct. |
| C. Changes made | References updated |
| D. Location | References, page 17 |

| | |
|---|---|
| Reviewer 3 | Dr. Ying Hu, Zhejiang University School of Medicine Women's Hospital |
| A. Comment 1 | It's better to provide more details of threatended preterm birth, such as the definition of uterine contractions, and the exclusion of preterm birth. |
| B. Response | The definition of threatened preterm birth used in the APOSTEL 8 study can be found in the methods section under study setting. |
| C. Changes made | For further clarification, we have added the definition in our abstract. |
| D. Location | Abstract, page 2 |

| | |
|---|---|
| Reviewer 3 | Dr. Ying Hu, Zhejiang University School of Medicine Women's Hospital |
| A. Comment | Page 5 line 44 "However, thus far only few randomised trials concerning tocolytic drug administration during pregnancy have performed longterm follow-up on child development." The author then listed two trials of your own research group, APOSTEL II trial and APOSTEL III trial. Are there no other studies on atosiban? |
| B. Response | At the time of the submission of this manuscript, we were unaware of any other studies concerning long-term follow-up of child outcomes after randomised trials concerning atosiban.<br><br>Recently, a large cohort study was published concerning long-term child outcomes after atosiban and nifedipine administration during pregnancy for threatened preterm birth based on premature prelabor rupture of membranes between 24 and 34 weeks gestation. |
| C. Changes made | Findings of this trial are added to our introduction. |
| D. Location | Introduction, page 6 |

| | |
|---|---|
| Reviewer 3 | Dr. Ying Hu, Zhejiang University School of Medicine Women's Hospital |

| | |
|---|---|
| A. Comment | How to consider covariates in statstical analysis, such as maternal age, educational level, etc. |
| B. Response | Since the current study concerns a follow-up study of a randomized trial, corrections on covariates will not be performed. In a randomized trial it is expected that baseline characteristics in both groups are comparable. We will provide a table with baseline characteristics where characteristics of the placebo group will be compared to the atosiban group.<br><br>Furthermore, we expect a 50% follow-up rate. To detect potential bias due to this follow-up rate, we will compare baseline characteristics of APOSTEL 8 follow-up participants to those lost to follow-up.<br><br>Our methods concerning these comparisons can be found in our method section under statistical analysis at page 12. |
| C. Changes made | None. |
| D. Location | - |

| Reviewer 4 | Dr. Zhaoxia Liang, Zhejiang University, Tulane University |
|---|---|
| A. Comment | Please clearly clarificate the reason for chosing threatened preterm birth between 30-34 weeks. |
| B. Response | In the Netherlands, before the start of the APOSTEL 8 study, tocolysis for threatened preterm birth from 24 to 34 weeks gestation was recommended by national guidelines. At the start of the trial, a nationwide adjustment of the tocolysis protocol had to occur to withhold tocolysis for threatened preterm birth and that it should only be administered in case of participation in the APOSTEL 8 study (if randomized in the atosiban group). It is difficult to discontinue an established treatment and physicians were hesitant. Therefore, the protocol was only adjusted for threatened preterm birth above 30 weeks gestation which is why a gestational age between 30 and 34 weeks of gestation was chosen in the APOSTEL 8 study. Since the current study is a follow-up study of the APOSTEL 8 study, we are bound to this gestation age at inclusion. |
| C. Changes made | None. |
| D. Location | - |

| Reviewer 4 | Dr. Zhaoxia Liang, Zhejiang University, Tulane University |
|---|---|
| A. Comment | Page 5 line 27 “no tocolytic drug has proven to be effective in reducing neonatal morbidity and mortality compared to a placebo.” Dose this mean that these studies included atosiban, or that there are currently no studies of atosiban research? |
| B. Response | With this line we refer to all studies performed on all six different groups of tocolytic agents. These studies also concern oxytocin receptor antagonists, including atosiban. |
| C. Changes made | An adjustment in this line is made for clarification. |

| | |
|---|---|
| D. | Introduction, page 5 line 27 |

| Reviewer 4 | Dr. Zhaoxia Liang, Zhejiang University, Tulane University |
|---|---|
| A. Comment | Page 7 line 21 “The primary outcome is a composite of adverse perinatal outcomes including perinatal mortality before discharge from hospital. Secondary outcomes include various infant and maternal outcomes. ” It’s better to provide more details of these outcomes. |
| B. Response | Thank you for your comment, we agree. |
| C. Changes made | We elaborated on the primary outcome of the APOSTEL 8 study. |
| D. Location | Method, page 7 line 21 |
